# Using a Hexagonal Mirror for Varying Light Intensity in the Measurement of Small-Angle Variation

**DOI:** 10.3390/s16081301

**Published:** 2016-08-16

**Authors:** Meng-Chang Hsieh, Jiun-You Lin, Chia-Ou Chang

**Affiliations:** 1 Institute of Applied Mechanics, National Taiwan University, No. 1, Sec. 4 Roosevelt Road, Taipei 10617, Taiwan; jang1985@msn.com; 2 Department of Mechatronics Engineering, National Changhua University of Education, 2, Shi-Da Road, Changhua 50074, Taiwan; jylin@cc.ncue.edu.tw; 3 College of Mechanical Engineering, Guangxi University, 100, University Road, Nanning 530004, China

**Keywords:** hexagonal mirror, small angle, Gaussian beam, laser beam shifter

## Abstract

Precision positioning and control are critical to industrial-use processing machines. In order to have components fabricated with excellent precision, the measurement of small-angle variations must be as accurate as possible. To achieve this goal, this study provides a new and simple optical mechanism by varying light intensity. A He-Ne laser beam was passed through an attenuator and into a beam splitter. The reflected light was used as an intensity reference for calibrating the measurement. The transmitted light as a test light entered the optical mechanism hexagonal mirror, the optical mechanism of which was created by us, and then it entered the power detector after four consecutive reflections inside the mirror. When the hexagonal mirror was rotated by a small angle, the laser beam was parallel shifted. Once the laser beam was shifted, the hitting area on the detector was changed; it might be partially outside the sensing zone and would cause the variation of detection intensity. This variation of light intensity can be employed to measure small-angle variations. The experimental results demonstrate the feasibility of this method. The resolution and sensitivity are 3 × 10^−40^ and 4 mW/° in the angular range of 0.6°, respectively, and 9.3 × 10^−50^ and 13 mW/° in the angular range of 0.25°.

## 1. Introduction

In the industry of precise manufacturing, the techniques of optical small-angle measurement have been extensively applied to the coordinates, alignment measurements of mechanical systems, precision laser drilling [1], and positioning. Measuring the small angle is critical in precision-positioning control application and mechanical calibration technology. Small-angle measurements in the semiconductor industry have been aimed at setting up equipment easily, and miniaturizing and attaining precise dimensions. Furthermore, some physical properties of materials also require precise angle measurements, such as the angular displacement measurement of test samples twisted by applied torques [2] for determining the relationship between shear stress and shear strain. 

Small-angle variations can be measured using various methods such as heterodyne interferometry [3], surface plasmon resonance [4], and image analysis [5]. However, the method of image analysis needs off-line image processing to obtain the measurement results. The method of surface plasmon resonance requires coating a thin metal film and the thermal effect [6] in the metal caused by the light source will deteriorate the measurement accuracy. The method of heterodyne interferometry has both polarization rotation and polarization mixing errors in the polarized components [7] which will cause measurement errors. In addition, to accurately measure angles, the prisms in total internal reflection and the mirrors in multiple light reflections [8,9,10] must demonstrate either superior right angles or parallelism; if the parallelism or right angle of a prism is not perfect, it will cause measurement errors [7]. There are excellent angular and displacement measurement methods [11,12,13] but these measurement structures are complicated. To facilitate fabricating components with high precision and to avoid the aforementioned measurement errors, this study proposes a new and simple optical mechanism, which is a hexagonal mirror that can parallel shift the light path and cause variations of light intensity at the detector. To reduce the effect of unstable source intensity on measurements, the intensity of the test light is divided by that of the reference light. Therefore, this system will present highly stable measurements [7,14]. This measurement of the light intensity variation can be utilized to evaluate small-angle variation. The proposed approach provides various advantages, such as a simple structure, high sensitivity, and high stability.

## 2. Materials and Methods 

### 2.1. Laser Beam Parallel Shifter 

**Figure 1:**
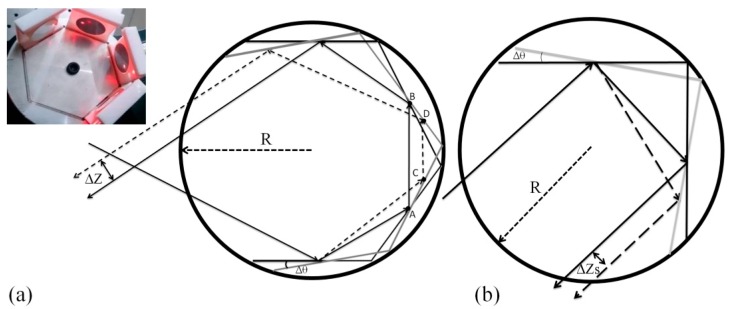
Parallel shift light paths before and after the angular variation. (**a**) Hexagonal mirror; (**b**) Square mirror.

A regular hexagon model was used in this study. Four mirrors were installed on four consecutive hexagonal sidewalls served as a laser beam parallel shifter as shown in Figure 1a. When a laser beam hit the first mirror and reflected off through four mirrors (Figure 1a, the solid line), the reflected light off the un-rotated hexagonal mirror was defined as the first light. When the hexagonal mirror rotated an angle Δ*θ*, the reflected light off the hexagonal mirror (Figure 1a, the dash line) was called the second light. After the second mirror reflection, the first light path of $\bar{AB}$ is parallel to the second path of $\bar{CD}$ as shown in Figure 1a; these two light paths are also parallel after the fourth mirror reflection (the geometric drawing based on the law of reflection, that is, the incident angle is equal to the reflective angle, it directly reveal that the two light paths are parallel through even times of reflections). The shifted distance between the first and second light is denoted by Δ*Z*. For hexagonal mirror, Δ*Z* is increased, as the reflection times do. In addition, the larger the angle variation Δ*θ* is, the greater the shifted distance Δ*Z* becomes, as shown in Figure 1a.

The characteristics of hexagonal mirror were utilized to create an angle sensor for measuring the rotation angle according to the distance between the parallel two reflective lights. For hexagonal mirror, the expression relating Δ*Z* and Δ*θ* is given by Equation (1),

(1) $$\Delta Z=2\sqrt{3}R\sin(\Delta \theta ),$$

which is derived in detail in the Supplementary Materials. The *R* in Equation (1) is the circumradius and equal to the side length of regular hexagon. Figure 1b shows the parallel shifted light path of square mirror before and after the angle variation. The relationship between Δ*Z* and Δ*θ* is derived as (see Supplementary Materials)

(2) ΔZs=2Rsin(Δθ)

By the comparison between Equations (1) and (2), it is found that, for the same radius of circumcircle R and same angle variation Δ*θ*, the hexagonal mirror has the greater shifted distance Δ*Z* than Δ*Zs* of the square mirror. Therefore, the hexagonal mirror provides higher angular sensitivity than the square mirror. At the same circumcircle size, the more the regular polygon number is, the better the sensitivity is, and the less the measurable range is.

**Figure 2:**
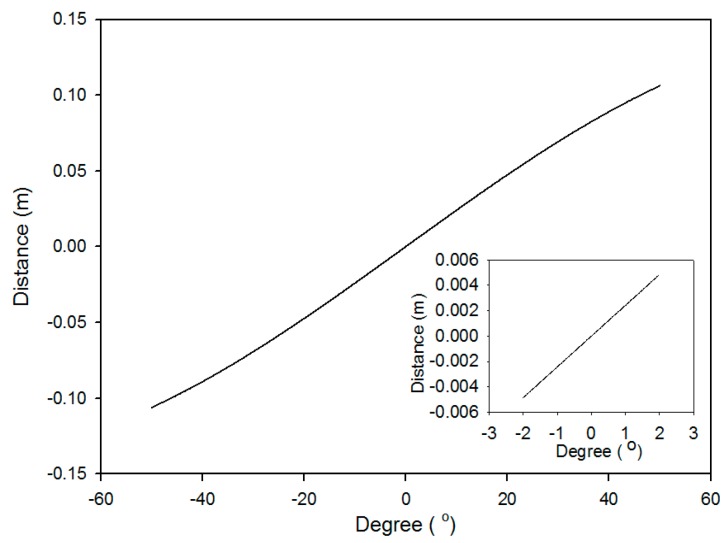
Relationship between Δ*θ* and Δ*Z*.

When the distance between these two lights had been measured, the variations of rotation angle would be determined by Equation (1), where the side-length of regular hexagon is equal to the radius R and was given 40 mm here. Figure 2 shows the simulation result using Equation (1) to demonstrate the relationship between the angle variation (Δ*θ*) and the shifted distance (Δ*Z*). The results indicate that the angle and distance variation are relatively linear within ±3°. To prevent the last reflective point being outside of the hexagonal mirror, the rotational angle was restricted within ±2°.

### 2.2. Laser Parallel Shift and Light Intensity Variation

**Figure 3:**
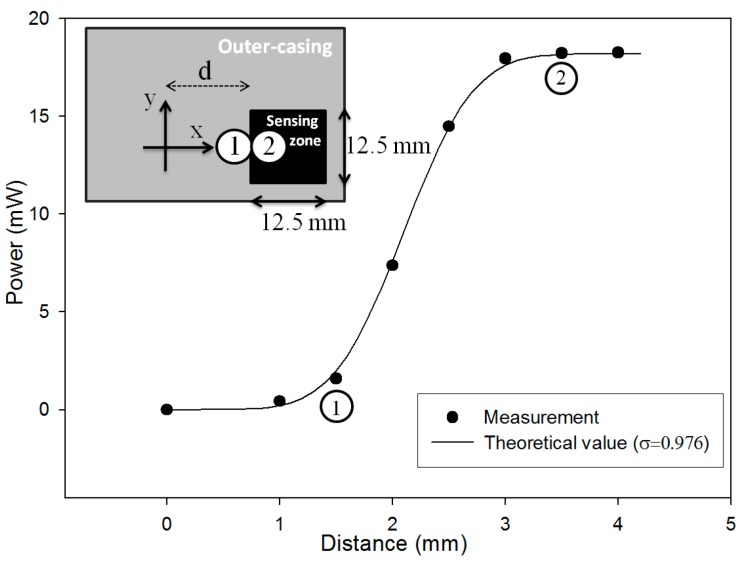
Light parallel shifts corresponding to light intensity variations.

The hexagonal mirror is an optical mechanism for detecting the angle variation by measuring the shifted distance. As the light beam was parallel shifted due to the angular motion of the mirror, the hitting point would advance from the outer-casing of power detector (NOVA II, Sensor size: 12.5 × 12.5 mm, OPHIR, Jerusalem, Israel) to its sensing zone, as shown in Figure 3; that is, the hitting point at the outer-casing boundary marked by ① moved to the point marked by ② located at the sensing zone. After ②, the Gaussian waveform of laser beam was completely inside the sensing zone, the intensity was saturated. The variation in intensity is particularly prominent in the vicinity of light beam crossing the boundary. Thus, that sensitive range was used as the sensing range for the detection.

### 2.3. The Definition of Laser Gaussian Distribution σ Value

The intensity distribution of the laser can be considered a Gaussian distribution [15,16,17] having its general form expressed as Equation (3):

(3) $$z=\sqrt{\frac{2}{\sigma^{2}\pi }e^{-\frac{2x^{2}}{\sigma^{2}}}}$$

In order to determine the value *σ* of laser Gaussian distribution, the laser light source was gradually displaced from 0 mm to 4.2 mm as shown in Figure 3. When the laser light source intensity is located at position c_i_ (the central position of laser spot) its intensity is expressed by Equation (4) as

(4) $$I(c_{i},\sigma )=I_{0}\frac{2}{\sigma^{2}\pi }\int_{d}^{d+12.5}\int_{-6.25}^{6.25}e^{\frac{2{\left(x-c_{i}\right)}^{2}}{\sigma^{2}}}e^{\frac{2y^{2}}{\sigma^{2}}dydx},$$

where *I_o_* represents the laser intensity and *d* is the distance from the origin of the coordinate to the left boundary of the sensing zone as shown in Figure 3. The upper and lower limits of the y integral (±6.25 mm) are determined by the size of power detector. Equation (4) shows that the change of laser spot position *c_i_* will alter the intensity *I*(*c_i_*, *σ*)*.*

To evaluate the undetermined value *σ*, the numerical approximation conducts the principle of minimizing the square root of sum of squared difference between the experimental measurement intensity (*m_i_*) and theoretical one *I*(*c_i_*, *σ*), which is expressed by Equation (5) as

(5) $$Error(\sigma )=\sqrt{\sum_{i=1}^{8}{\left(I(c_{i},\sigma )-m_{i}\right)}^{2}}$$

**Figure 4:**
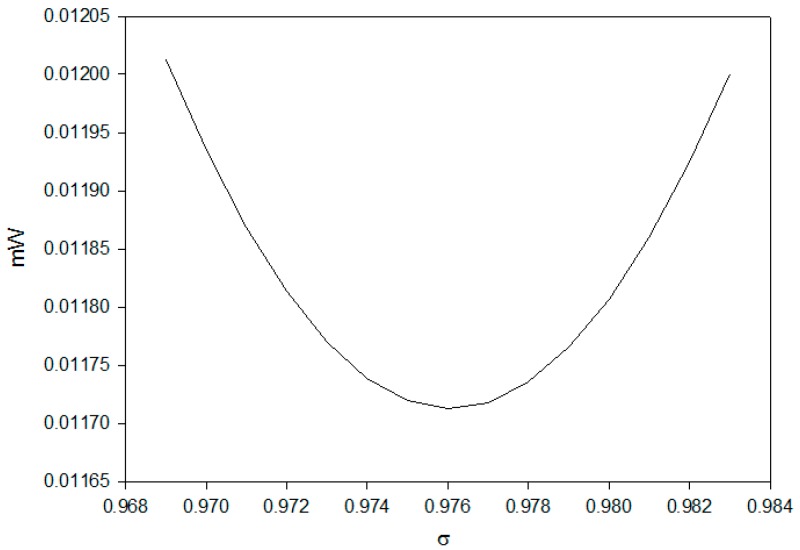
The distribution of error between the experimental and theoretical values in different *σ*.

The error function Equation (5) was calculated and plotted versus *σ* in Figure 4.

The σ was found to be 0.976 according to the minimum of error function shown in Figure 4. The theoretical intensity based on *σ* *=* 0.976 matches very well with the measured one as shown in Figure 3.

## 3. Experimental Setup

**Figure 5:**
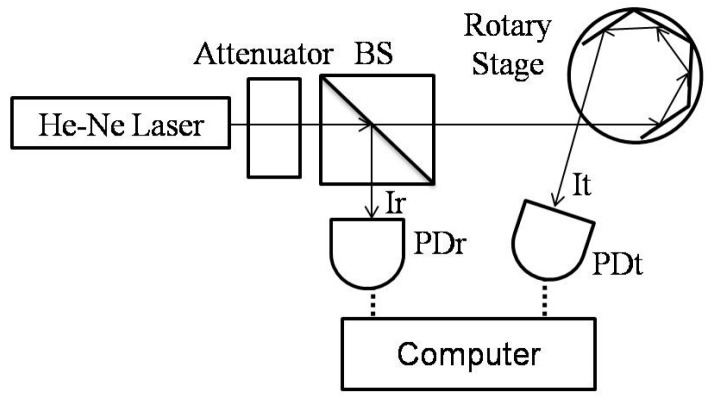
Experimental architecture.

In the proposed experimental scheme, the laser light source (25LHP925-249, Melles Griot, Carlsbad, CA, USA) was fitted with an attenuator as shown in Figure 5. When the rotary stage (SGSP-60YAW, Opto Sigma, Santa Ana, CA, USA) was rotated in a small angle and the laser beam was shifted, the hitting area on the detector was changed and might be partially outside the sensing zone, it would cause the variation of detection intensity, as shown in Figure 3 and Figure 5. To avoid measurement errors caused by the inclined hexagonal mirror and the unstable laser intensity, the position calibration of the mirror was performed and a beam splitter (BS) was used to split a reference light from the laser, respectively. The reference light intensity (Ir) is detected by the reference power detector (PDr) and the test light intensity (It) is detected by the test power detector (PDt) (for intensity stability the resolution of the power detector was chosen to be 1 µW in this study). Both It and Ir are error-prone due to a noise and unstable source (i.e., variations in laser intensity relative to the duration of usage), the expression of It/Ir would be used instead for the purpose of noise-elimination. Therefore, this system will present highly stable measurements.

## 4. Results and Discussions

### 4.1. Experimental Results

**Figure 6:**
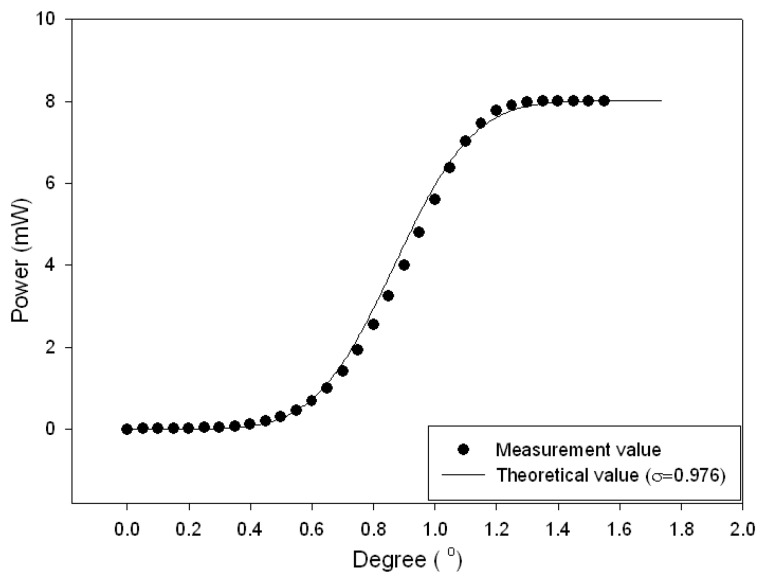
Intensity versus angle variation (Power (mW) − Δθ (°)).

This study proposed a method for measuring small-angle variations by detecting the variations of light intensity. The rotation of the hexagonal mirror by a small angle Δ*θ* causes the laser beam to be parallel shifted by Δ*Z* (given in Equation (1)), and this affects the intensity (given in Equation (4)) of the light entering the PDt; therefore, the rotation angle *Δθ* can be determined by the variations of the intensity. Figure 6 shows the experimental results of the test light intensity (It). Considerable variation (high sensitivity) in light intensity is observed between 0.6° and 1.2°, and there is especially good linearity in the range of 0.75° and 1.1°; therefore, the ranges are used for measuring small-angle variations. Figure 6 shows that the theoretical results are consistent with the measured ones.

### 4.2. The Measurement of Linearity and Reference Light Correction

**Figure 7:**
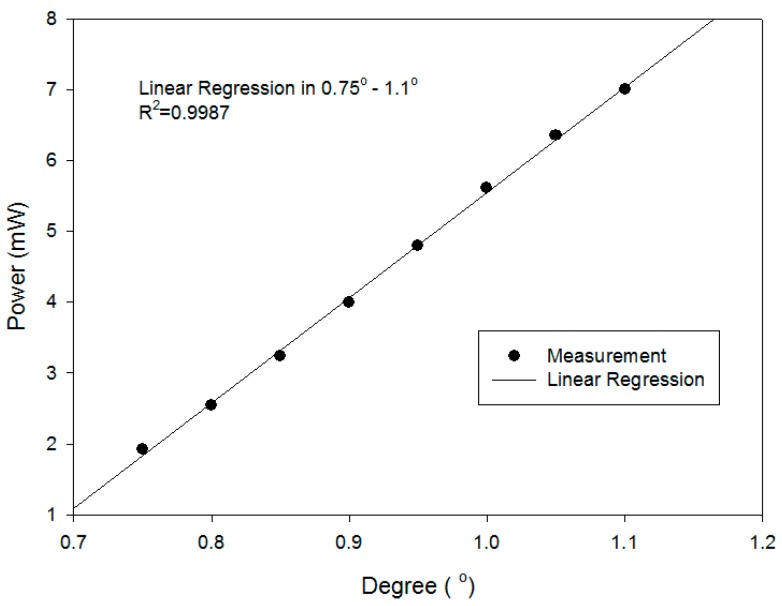
The linear regression of intensity versus angle variation.

Figure 7 depicts the relationship between the angle variation and the intensity in the highly sensitive region of Figure 6. The highly sensitive region between 0.75° and 1.1° was adopted. In Figure 7, a curve of the linear regression showed the linearity of the measurement was high (*R*^2^ = 0.9987).

**Figure 8:**
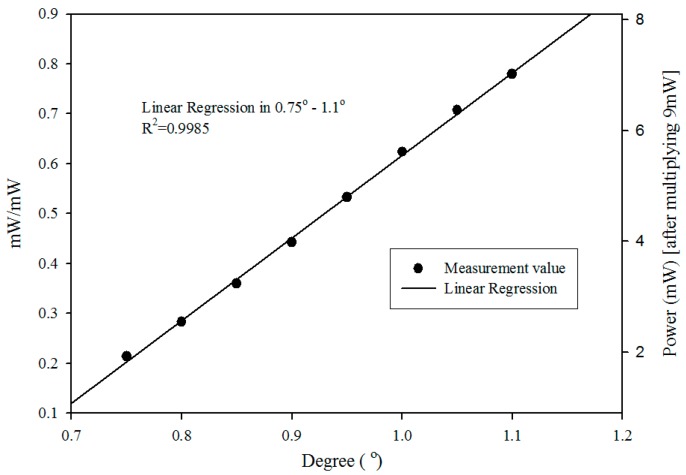
Test light intensity divided by the reference light intensity for determining the small-angle variation and plotted against the linear regression curve.

This reference correction system (Figure 5) enables the simultaneous detection of the test and reference lights’ intensities. The correction is done by dividing the test light’s intensity by the reference light’s intensity. The left vertical axis of Figure 8 is the value of It/Ir. The right vertical axis is the ratio It/Ir multiplied by the stable-state intensity of the reference light (9 mW was the stable-state intensity in this experiment), which is added for the clarity of the physical quantity. Figure 8 shows that, after the reference correction, there was still good linearity (the linear regression, *R*^2^ = 0.9985).

### 4.3. The Sensitivity and Resolution

**Figure 9:**
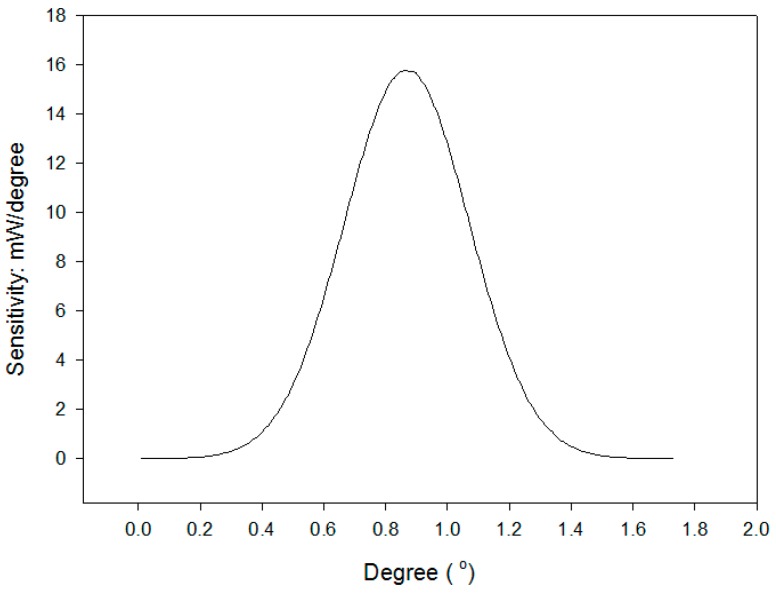
The sensitivity of experimental architecture (*σ* is 0.976).

The sensitivity (*S =* Δ*power/*Δ*θ*) of this architecture is superior to 4 (mW/°) in the range of 0.6° to 1.2° and to 13 (mW/°) in the range of 0.75° to 1.0° as shown in Figure 9. If the laser has good intensity and the detector is of a high resolution, the sensitivity and resolution for the measurement of angle variations can be improved. The relationship between Δ*θ* and Δ*power* can be expressed as follows:

(6) $$\Delta \theta =\left(\frac{\partial \theta }{\partial power(\theta )}\right)\Delta power$$

As revealed in Equation (6), Δ*θ* can be estimated by measuring Δ*power*. In addition, the resolution of this system [14,18,19] can be defined as

(7) $$\Delta \theta_{err}=\left|\frac{\Delta power_{err}}{\Delta power}\right|\Delta \theta =\Delta power_{err}\frac{1}{S}$$

**Figure 10:**
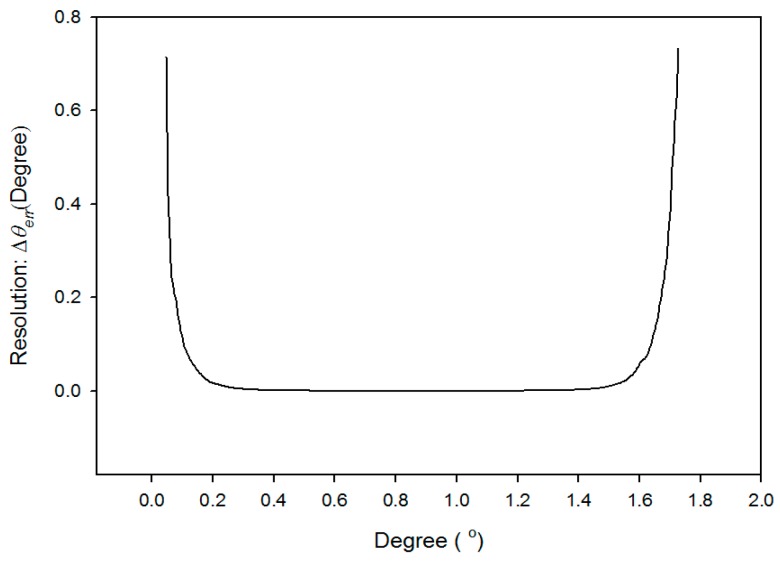
The resolution of experimental architecture (*σ* is 0.976).

The resolution of the power detector in this study is 1 µW. The maximum and minimum standard deviations of the signal error are 2.7 μW and 1.4 nW. The average standard deviation of the signal error is 1.2 μW. The resolution levels Δ*θ_err_* are found from Figure 10 to be superior to 3 × 10^−^^40^ in the ranges from 0.6° to 1.2° and 9.3 × 10^−^^50^ in the ranges from 0.75° to 1.0°, respectively. They can be evaluated by the reciprocal of the sensitivity (S) (Figure 9) and Equation (7) [19,20] at the average standard deviation of the signal error Δ*power_err_* = 1.2 µW.

## 5. Conclusions

The proposed hexagonal mirror architecture can be used to measure small-angle variations in a new and simple method. Through the observation of the light intensity variation, the angle variation can be determined. The proposed design can be applied to detect reference light intensity and to eliminate the factors caused by the laser intensity variation, which would affect the precision of the measurement. The proposed structure is easy to assemble, low-cost, and highly stable. Based on these advantages, it can be effectively used to measure small-angle variations. The resolution and sensitivity are 3 × 10^−40^ and 4 mW/° in the angle interval of 0.6°, and 9.3 × 10^−50^ and 13 mW/° in the interval of 0.25°, respectively.

## Supplementary Materials

The supplementary materials are available online at http://www.mdpi.com/1424-8220/16/8/1301/s1, Hexagonal Mirror, Square Mirror.
