# Supplementary material for: Using a Hexagonal Mirror for Varying Light Intensity in the Measurement of Small-Angle Variation
Source: Sensors (Basel). 2016 Aug 16;16(8):1301. doi: 10.3390/s16081301 (PMC5017466; doi:10.3390/s16081301)
Supplement: Supplementary file 1 [file sensors-16-01301-s001.pdf]

Hexagonal Mirror

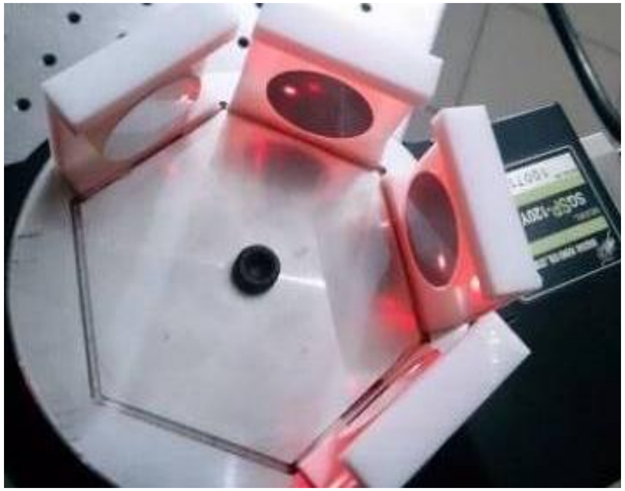

When the hexagonal mirror was rotated by a small angle(Δθ), the laser beam was parallel shifted(ΔZ).

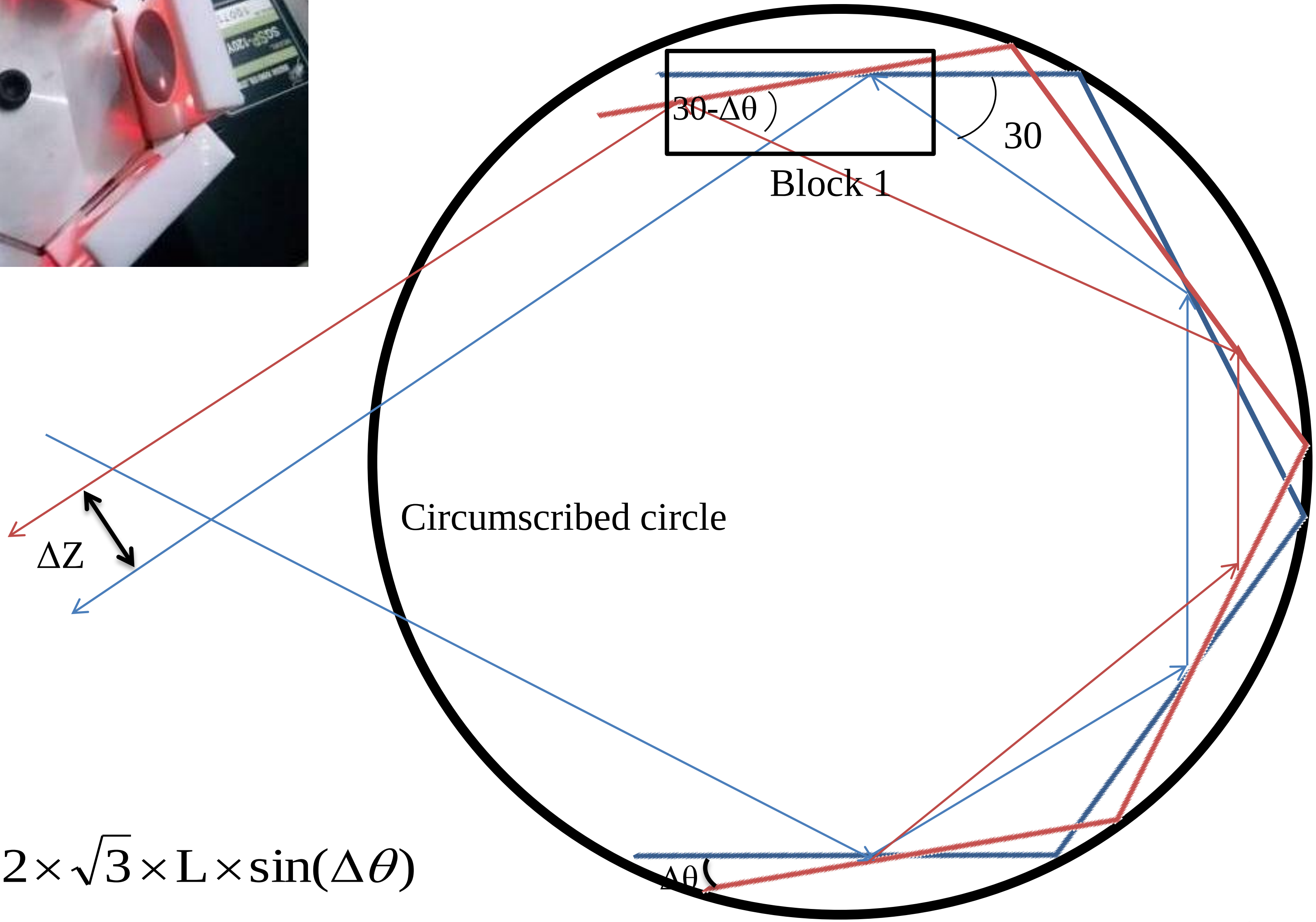

$$\Delta z = 2 \times \sqrt{3} \times L \times \sin(\Delta \theta)$$

Block 1

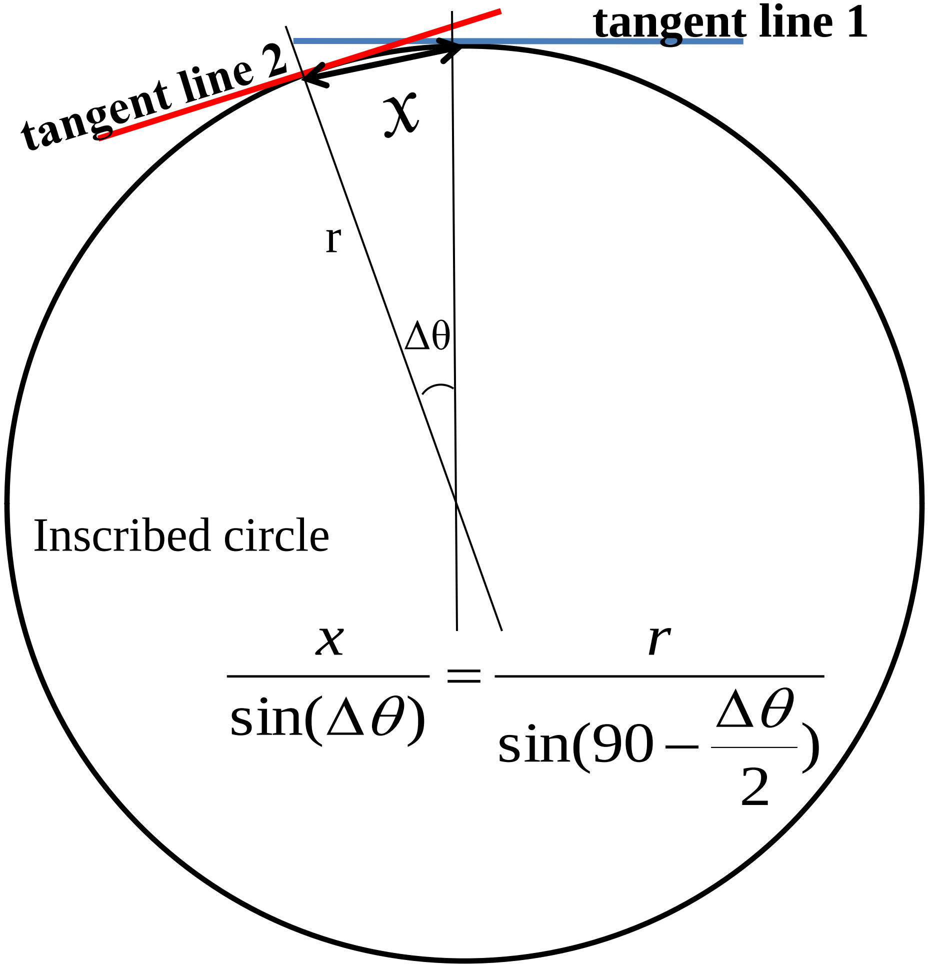

$$\frac{x}{\sin(\Delta\theta)} = \frac{r}{\sin(90 - \frac{\Delta\theta}{2})}$$

x=the distance of two tangent line

According to sine rule, we can find w.

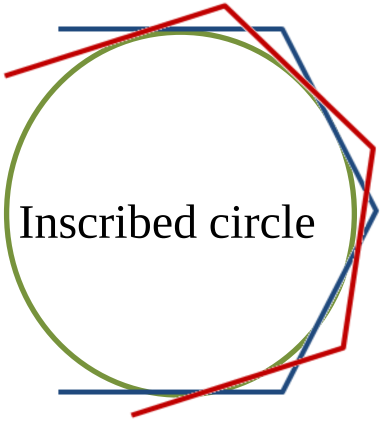

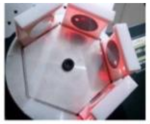

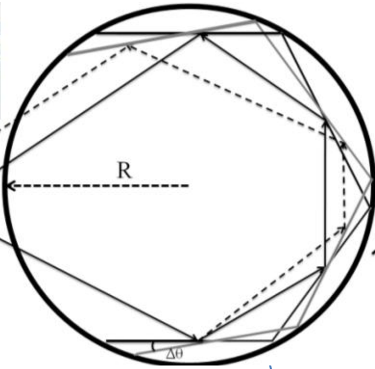

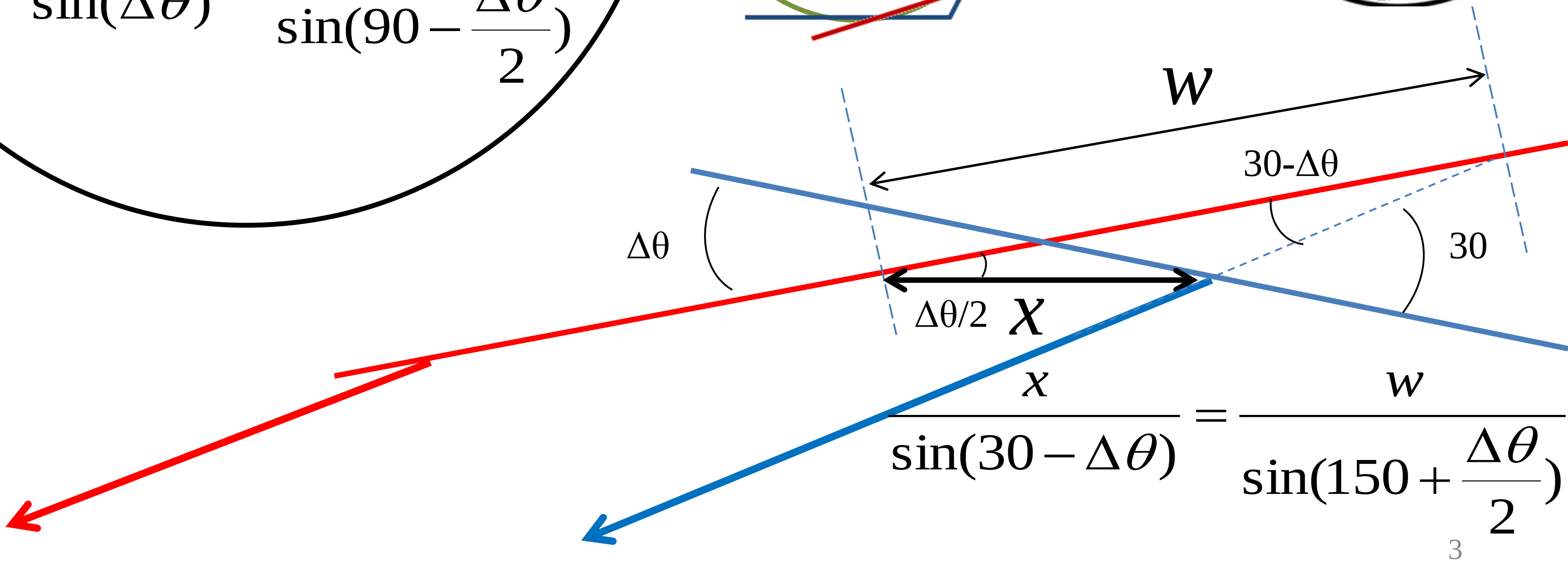

$$\frac{x}{\sin(30 - \Delta\theta)} = \frac{w}{\sin(150 + \frac{\Delta\theta}{2})}$$

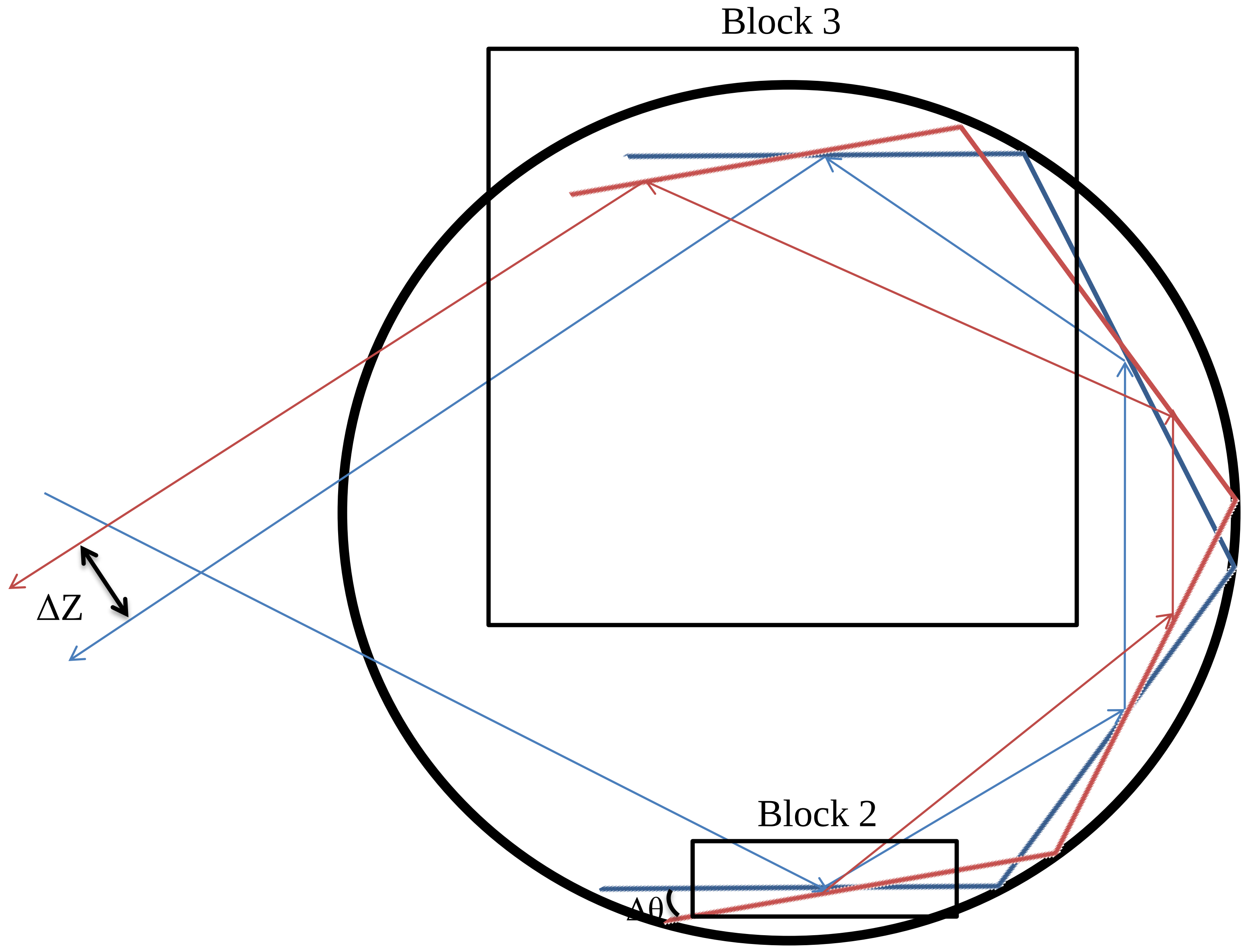
Block 3
ΔZ
Block 2
Δθ

Block 4

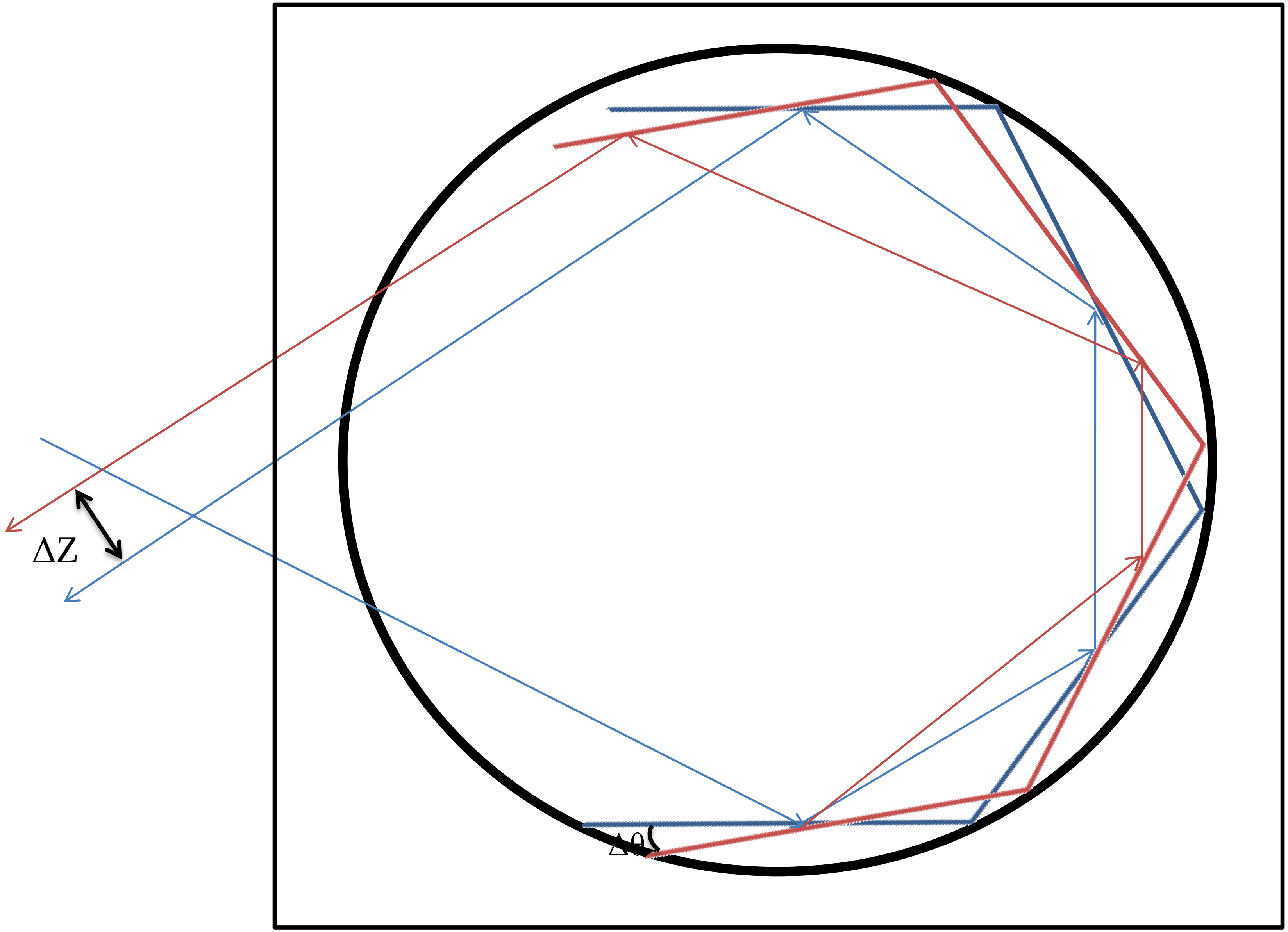

$$\frac{x}{\sin(\Delta\theta)} = \frac{r}{\sin(90 - \frac{\Delta\theta}{2})}$$

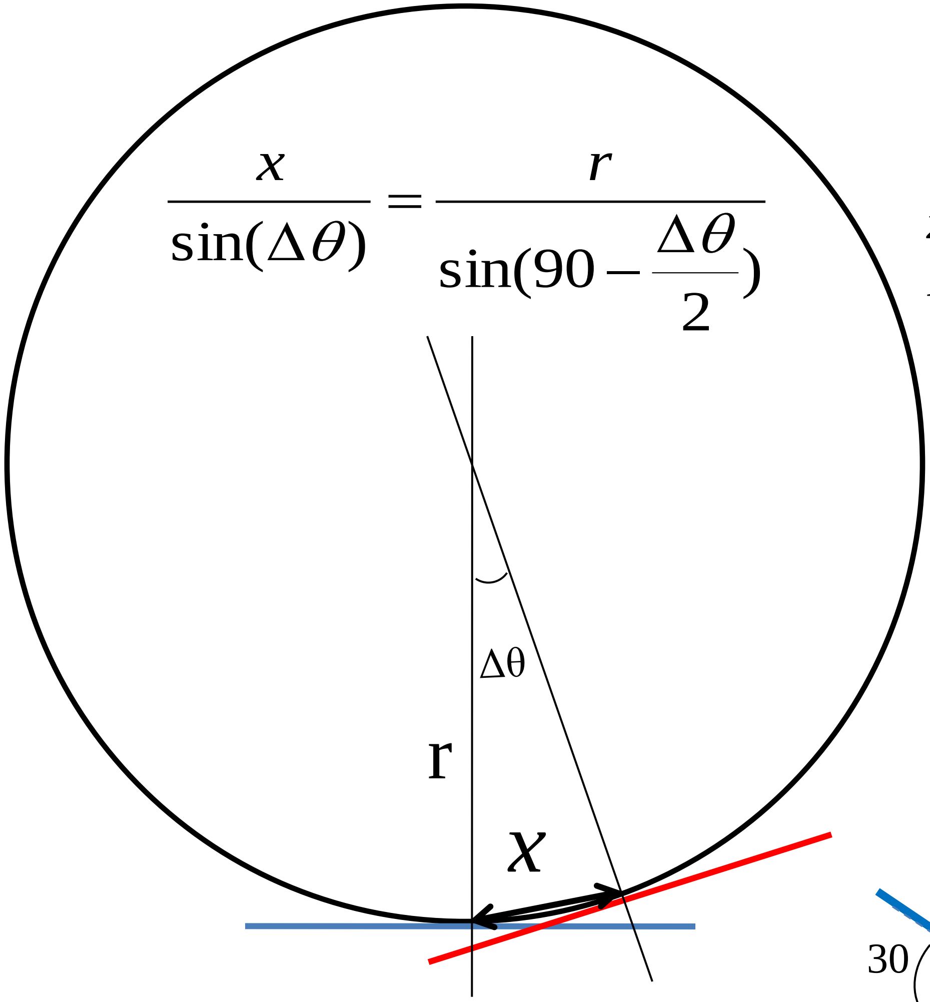

x=the distance of two tangent line
According to sine rule, we can find *y*.

r: The radius of inscribed circle.
r will mention in the next page.

$$\frac{x}{\sin(150 - \Delta\theta)} = \frac{y}{\sin(30 + \frac{\Delta\theta}{2})}$$

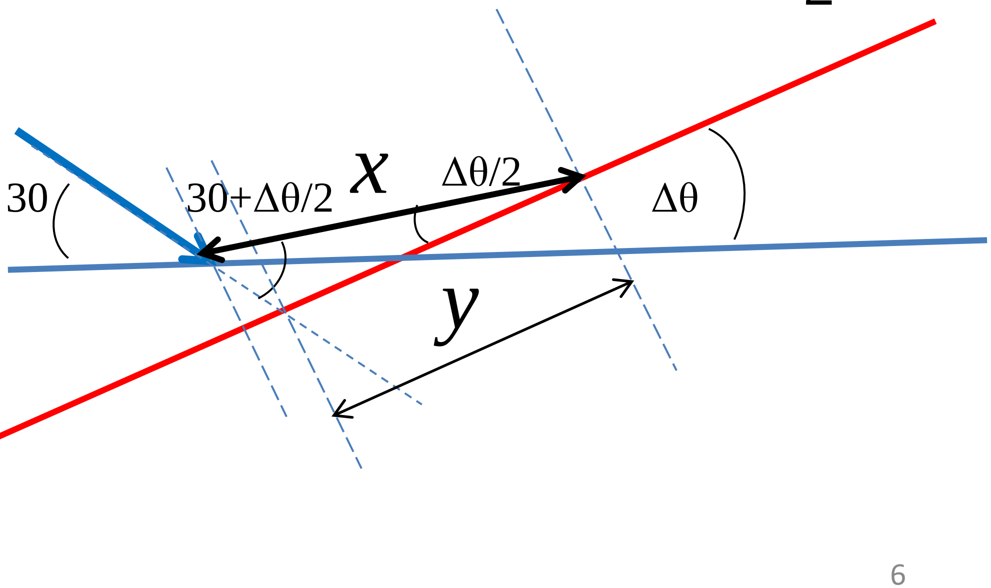

Block 3: R is the radius of circumscribed circle.

R is equal to the side length of hexagonal mirror (L).

$$r = \frac{\sqrt{3}}{2} R$$

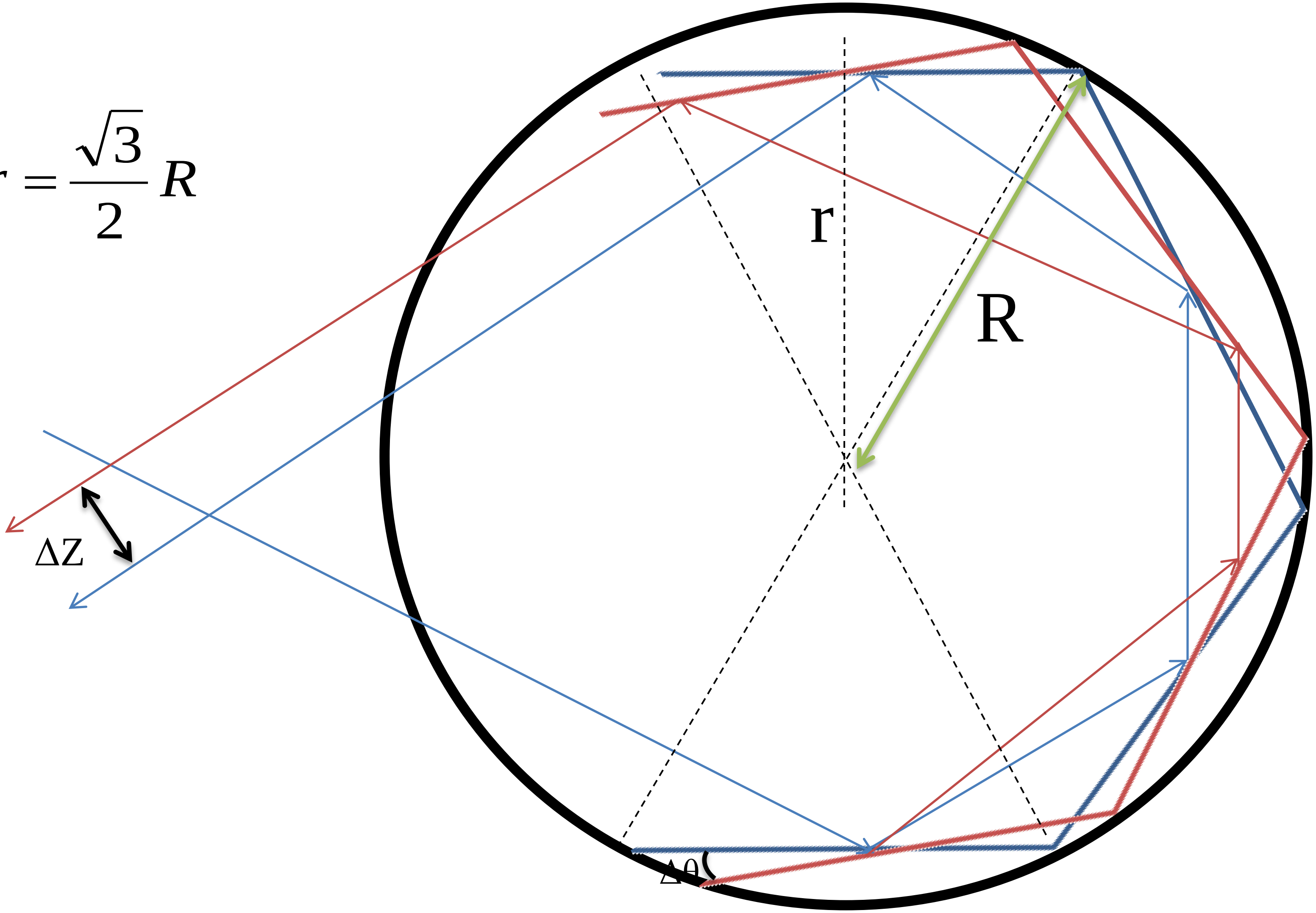

Block 4

$$\frac{y + R/2}{\sin(30-\Delta\theta)} = \frac{d1}{\sin(30+\Delta\theta)}$$

$$\frac{R - d1}{\sin(30+\Delta\theta)} = \frac{d2}{\sin(30-\Delta\theta)}$$

$$\frac{R - d2}{\sin(30-\Delta\theta)} = \frac{d3}{\sin(30+\Delta\theta)}$$

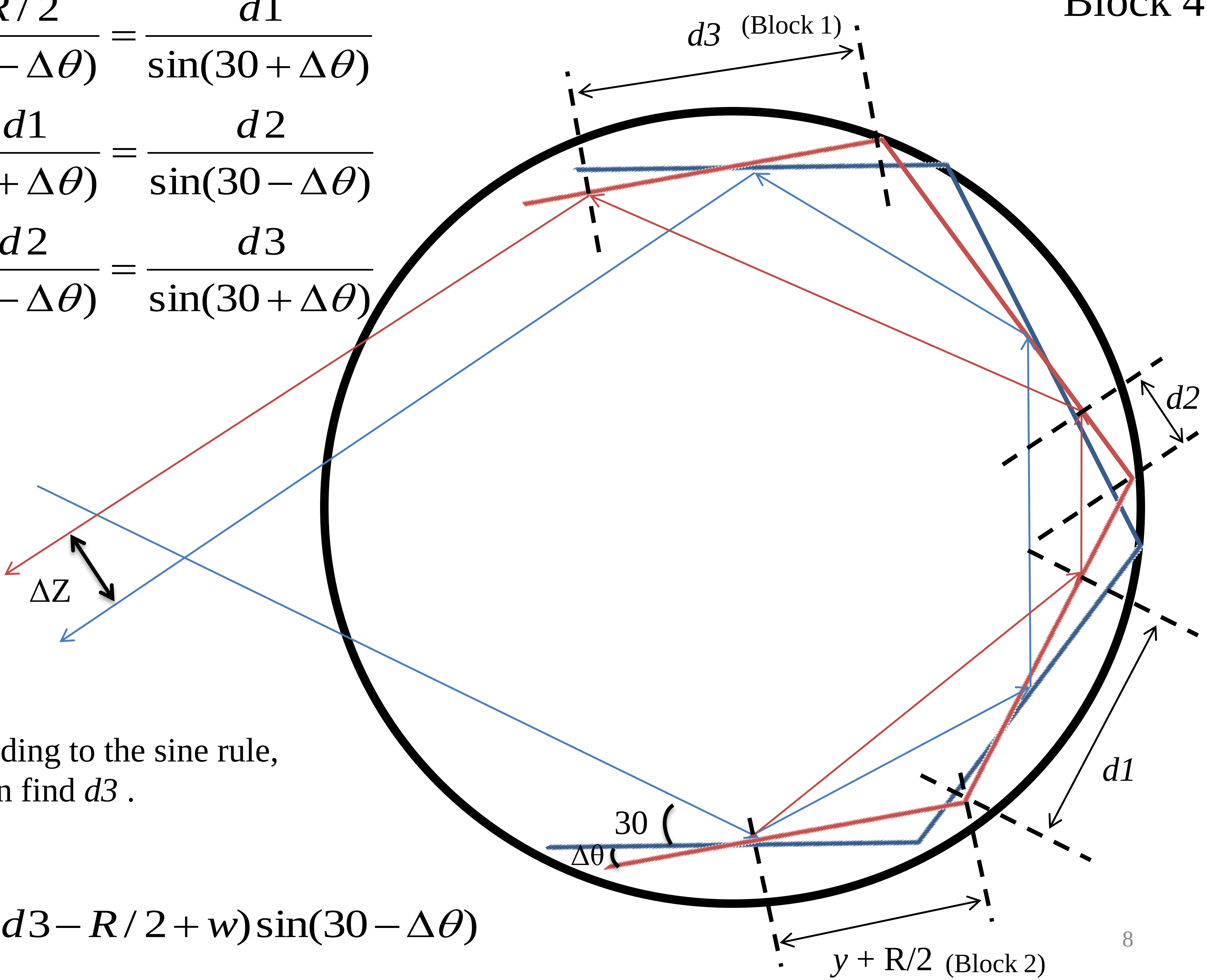

According to the sine rule, we can find *d3* .

$$\Delta z = (d3 - R/2 + w)\sin(30-\Delta\theta)$$

$$r = \frac{\sqrt{3}}{2} R$$

$$\frac{x}{\sin(\Delta\theta)} = \frac{r}{\sin(90 - \frac{\Delta\theta}{2})}$$

$$\frac{x}{\sin(30 - \Delta\theta)} = \frac{w}{\sin(150 + \frac{\Delta\theta}{2})}$$

w

Substituting *d3* and *w* into Δz.

$$\Delta z = (d3 - R/2 + w)\sin(30 - \Delta\theta)$$

$$\frac{x}{\sin(150 - \Delta\theta)} = \frac{y}{\sin(30 + \frac{\Delta\theta}{2})}$$

$$\frac{y + R/2}{\sin(30 - \Delta\theta)} = \frac{d1}{\sin(30 + \Delta\theta)}$$

$$\frac{\mathrm{R} - d1}{\sin(30 + \Delta\theta)} = \frac{d2}{\sin(30 - \Delta\theta)}$$

$$\frac{\mathrm{R} - d2}{\sin(30 - \Delta\theta)} = \frac{d3}{\sin(30 + \Delta\theta)}$$

$d3$

$$d3=\left\{\mathrm{R}-\left[\mathrm{R}\frac{\sin(30-\Delta\theta)}{\sin(30+\Delta\theta)}-\left(\frac{\sqrt{3}R\sin(\Delta\theta)\sin(30+\frac{\Delta\theta}{2})}{2\sin(180-\Delta\theta-30)\cos(\frac{\Delta\theta}{2})}+\frac{\mathrm{R}}{2}\right)\right]\right\}\times\frac{\sin(30+\Delta\theta)}{\sin(30-\Delta\theta)}$$

$$w=\frac{\sqrt{3}R\sin(\Delta\theta)\sin(150+\frac{\Delta\theta}{2})}{2\cos(\frac{\Delta\theta}{2})\sin(30-\Delta\theta)}$$

***d3* and *w* Substituted into**

$$\Delta z=(d3-R/2+w)\sin(30-\Delta\theta)$$

Calculation A (next page) R=L $(R:\mathrm{Circumradius})$

$$\Delta z=2\sqrt{3}\,\mathrm{L}\sin(\Delta\theta)$$

Calculation A

$$d3=\left\{R-\left[R\frac{sin(30-\Delta\theta)}{sin(30+\Delta\theta)}-\left(\frac{\sqrt{3}R\,sin(\Delta\theta)sin(30+\frac{\Delta\theta}{2})}{2\,sin(180-\Delta\theta-30)cos(\frac{\Delta\theta}{2})}+\frac{R}{2}\right)\right]\right\}\times\frac{sin(30+\Delta\theta)}{sin(30-\Delta\theta)}=\frac{3R}{2}\frac{sin(30+\Delta\theta)}{sin(30-\Delta\theta)}-R+\frac{\sqrt{3}R\,sin(\Delta\theta)sin(30+\frac{\Delta\theta}{2})sin(30+\Delta\theta)}{2\,sin(150-\Delta\theta)cos(\frac{\Delta\theta}{2})sin(30-\Delta\theta)}$$

$$w=\frac{\sqrt{3}R\,sin(\Delta\theta)sin(150+\frac{\Delta\theta}{2})}{2\,cos(\frac{\Delta\theta}{2})sin(30-\Delta\theta)}$$

$$\Delta z=(d3-R/2+w)sin(30-\Delta\theta)$$

Substituting *d3* and w into Δz.

$$\Delta z=\left(\frac{3R}{2}\frac{sin(30+\Delta\theta)}{sin(30-\Delta\theta)}-R+\frac{\sqrt{3}R\,sin(\Delta\theta)sin(30+\frac{\Delta\theta}{2})sin(30+\Delta\theta)}{2\,sin(150-\Delta\theta)cos(\frac{\Delta\theta}{2})sin(30-\Delta\theta)}-R/2+\frac{\sqrt{3}R\,sin(\Delta\theta)sin(150+\frac{\Delta\theta}{2})}{2\,cos(\frac{\Delta\theta}{2})sin(30-\Delta\theta)}\right)sin(30-\Delta\theta)$$

$$\Delta z=\frac{3R}{2}sin(30+\Delta\theta)-\frac{3R}{2}sin(30-\Delta\theta)+\frac{\sqrt{3}R\,sin(\Delta\theta)}{2\,cos(\frac{\Delta\theta}{2})}\left[sin(30+\frac{\Delta\theta}{2})+sin(150+\frac{\Delta\theta}{2})\right];\ sin(30+\frac{\Delta\theta}{2})+sin(150+\frac{\Delta\theta}{2})=2\,sin(30)cos(\frac{\Delta\theta}{2})$$

$$\Delta z=\frac{3R}{2}\times 2\,cos(30)sin(\Delta\theta)+\frac{\sqrt{3}R\,sin(\Delta\theta)}{2}=\frac{3\sqrt{3}R}{2}\times sin(\Delta\theta)+\frac{\sqrt{3}R\,sin(\Delta\theta)}{2}$$

$$\Delta z=2\sqrt{3}R\,sin(\Delta\theta)$$

Square Mirror

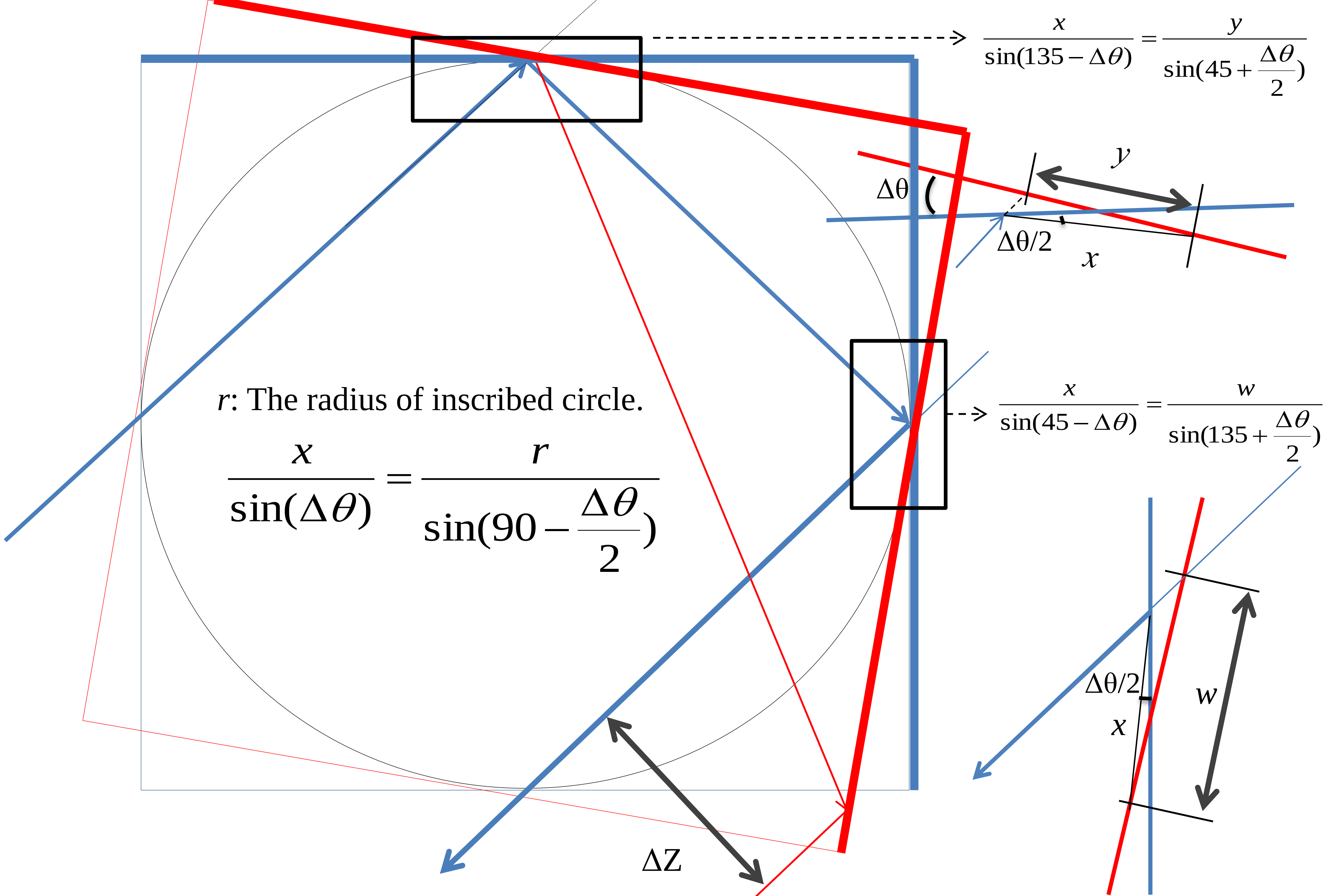

x=the distance of two tangent line

According to sine rule, we can find *y* and *w*.

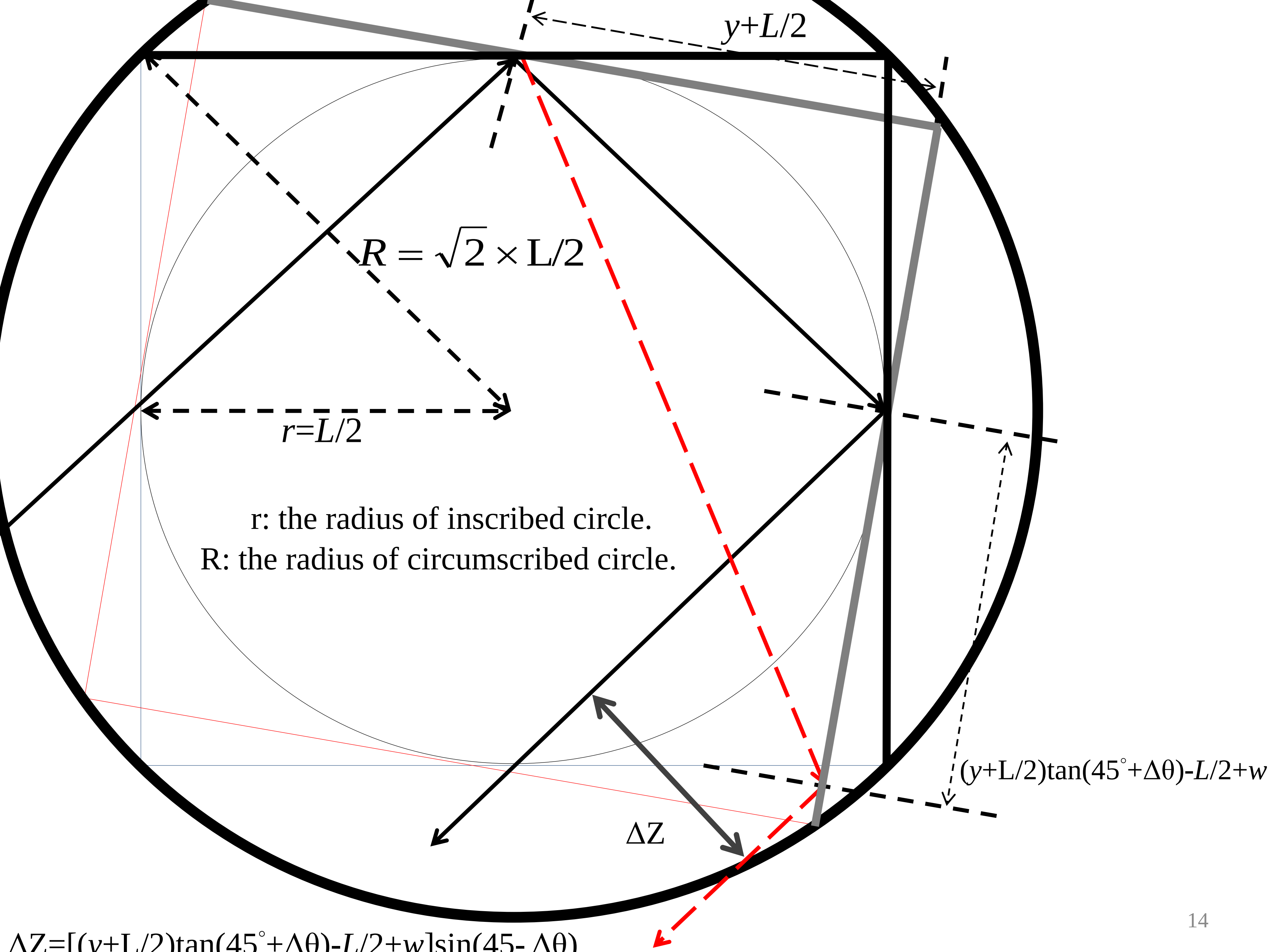

$\Delta Z = [(y + L/2)\tan(45^\circ + \Delta\theta) - L/2 + w]\sin(45 - \Delta\theta)$

$$x=\frac{r\sin(\Delta\theta)}{\sin(90-\frac{\Delta\theta}{2})},\ y=\frac{x\sin(45+\Delta\theta/2)}{\sin(135-\Delta\theta)}=\frac{r\sin(\Delta\theta)\sin(45+\Delta\theta/2)}{\sin(90-\frac{\Delta\theta}{2})\sin(135-\Delta\theta)},\ w=\frac{x\sin(135+\Delta\theta/2)}{\sin(45-\Delta\theta)}=\frac{r\sin(\Delta\theta)\sin(45-\Delta\theta/2)}{\sin(90-\frac{\Delta\theta}{2})\sin(45-\Delta\theta)}$$

Substituting *x, y and w into* Δz.

$$\Delta Z=[(y+L/2)\tan(45+\Delta\theta)-L/2+w]\sin(45-\Delta\theta);(L/2=r)$$

The individual multiplication of each term.

$$y\tan(45+\Delta\theta)\sin(45-\Delta\theta)=\frac{r\sin(\Delta\theta)\sin(45+\Delta\theta/2)}{\sin(90-\frac{\Delta\theta}{2})\sin(135-\Delta\theta)}\frac{\sin(45+\Delta\theta)}{\cos(45+\Delta\theta)}\sin(45-\Delta\theta)=\frac{r\sin(\Delta\theta)\sin(45+\Delta\theta/2)}{\cos(\frac{\Delta\theta}{2})}\text{......}(1)$$

$$L/2\tan(45+\Delta\theta)\sin(45-\Delta\theta)=r\frac{\sin(45+\Delta\theta)}{\cos(45+\Delta\theta)}\sin(45-\Delta\theta)\text{......}(2)$$

$$-L/2\sin(45-\Delta\theta)=-r\sin(45-\Delta\theta)\text{......}(3)$$

$$w\sin(45-\Delta\theta)=\frac{r\sin(\Delta\theta)\sin(45-\Delta\theta/2)}{\sin(90-\frac{\Delta\theta}{2})\sin(45-\Delta\theta)}\sin(45-\Delta\theta)=\frac{r\sin(\Delta\theta)\sin(45-\Delta\theta/2)}{\cos(\frac{\Delta\theta}{2})}\text{......}(4)$$

$$(2)+(3):r\sin(45+\Delta\theta)-r\sin(45-\Delta\theta)=r2\cos 45\sin(\Delta\theta)\text{......}(5)$$

$$(1)+(4):\frac{r\sin(\Delta\theta)}{\cos(\frac{\Delta\theta}{2})}(\sin(45+\Delta\theta/2)+\sin(45-\Delta\theta/2))=\frac{r\sin(\Delta\theta)}{\cos(\frac{\Delta\theta}{2})}2\sin 45\cos(\frac{\Delta\theta}{2})=r2\sin 45\sin(\Delta\theta)\text{......}(6)$$

$$(5)+(6):r2\cos 45\sin(\Delta\theta)+r2\sin 45\sin(\Delta\theta)=2r\sin(\Delta\theta)(\cos 45+\sin 45)=2\sqrt{2}r\sin(\Delta\theta)\ \ (r:\text{Inscribed circle radius})$$

$$\Delta Z=2\sqrt{2}r\sin(\Delta\theta)=2R\sin(\Delta\theta)\quad(\sqrt{2}r=R)(R:\text{Circumradius});$$

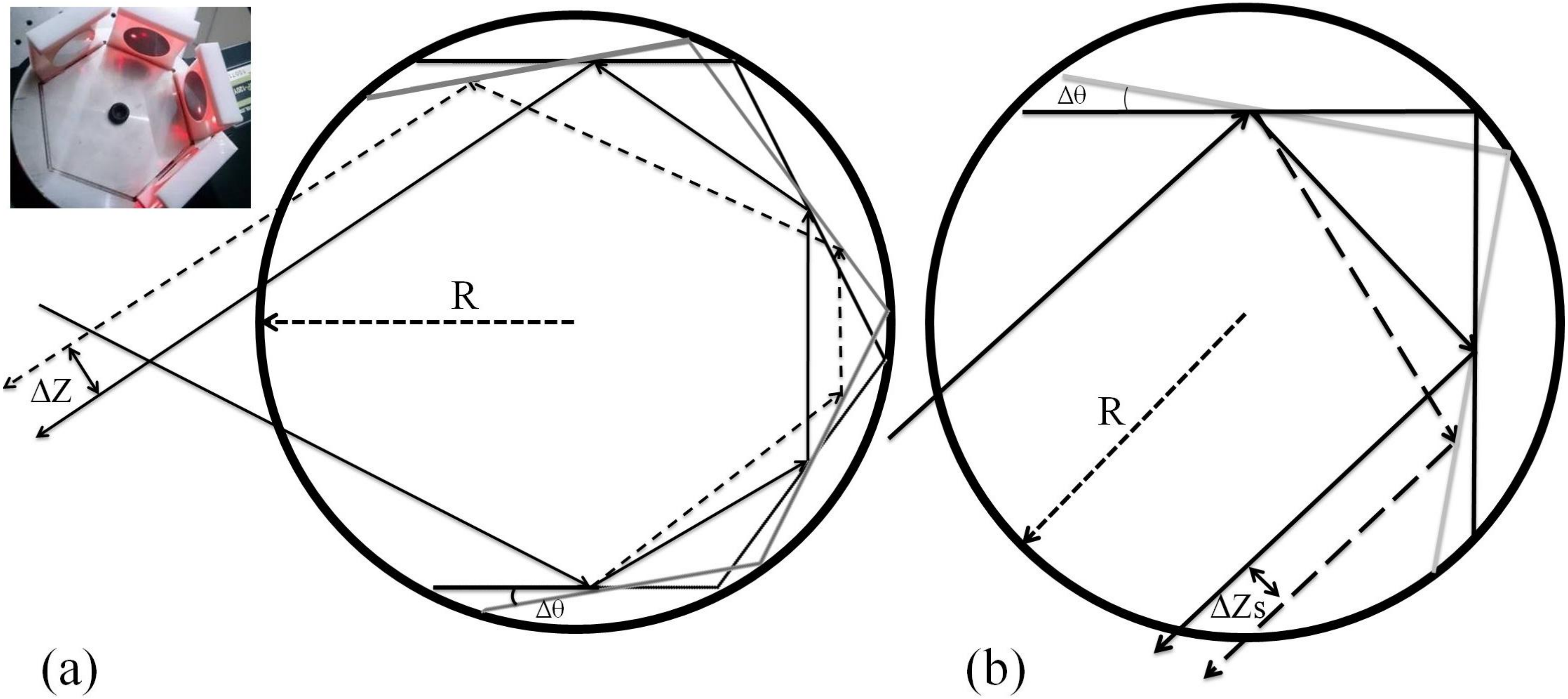
R
ΔZ
Δθ
(a)
Δθ
R
ΔZs
(b)
